# Exploiting temporal relationships in the prediction of mortality

**DOI:** 10.1016/S2589-7500(20)30056-X

**Published:** 2020-03-12

**Authors:** Christopher V Cosgriff, Leo Anthony Celi

**Affiliations:** MIT Critical Data, Laboratory for Computational Physiology, Massachusetts Institute of Technology, Cambridge, MA 02139, USA; Department of Medicine, Hospital of the University of Pennsylvania, Philadelphia, Pennsylvania, USA; MIT Critical Data, Laboratory for Computational Physiology, Massachusetts Institute of Technology, Cambridge, MA 02139, USA; and Division of Pulmonary Critical Care and Sleep Medicine, Beth Israel Deaconess Medical Center, Boston, MA, USA

Given a set of physiological signals and a selection of clinical history, what is the probability that a patient in the intensive care unit (ICU) will die either at the hospital or within a few months of discharge? As a predictive challenge, this question is broached countless times per year. The forecast probability of ICU mortality has various uses: it can inform clinical decision making and conversations with families regarding goals of care, serve as a way to adjust for case-mix or severity in inferential models, and allow departments to benchmark performance. In *The Lancet Digital Health*, Hans-Christian Thorsen-Meyer and colleagues apply deep learning to this task.^1^ More specifically, they train a long short-term memory (LSTM) neural network on physiological, laboratory, and clinical data to provide hourly predictions of 90-day mortality on an ICU cohort derived from large electronic health record databases in Denmark. They achieve discriminative performance that improves over the course of the patient’s ICU stay, and show the utility of an approach to make model predictions more interpretable.

Recently, Fritz and colleagues developed convolutional neural networks and LSTM-based models for a similar task: the prediction of 30-day post-operative mortality.^2^ In that case, as in the work by Thorsen-Meyer and colleagues, the main difference between traditional modelling approaches and deep learning is automatic feature extraction; for instance, instead of providing the model with patients’ median heart rate over 24 h, these models can take as input 24 hours’ worth of heart rate recordings at an arbitrary sampling frequency—or perhaps even a weeks’ worth—and learn complex relationships between these data and the outcome.^3^ Whether the dynamics of various physiological signals and laboratory data over time suggest an underlying model of chronic illness or response to treatment, these neural networks nevertheless learn complex relationships directly from data without the need for a human to engineer them.

As would be expected, the predictive performance of Thorsen-Meyer and colleagues’ model improves as it receives increasing amounts of data over the ICU stay. Considering that health-care systems and professionals collect a myriad of data on a patient each day, the authors’ choice to continually update their predictions as daily data accrues shows an clear way in which the data we collect could be put to better use. Thorsen-Meyer and colleagues go on to show that the relative importance of various input features in predicting survival or non-survival changes over the course of the ICU stay; their visual representation of these fluctuations is perhaps their most interesting contribution.

This work adds to the growing literature on the creative ways in which deep learning approaches can use the large swathes of health data collected daily to inform clinical decision making in a way that prospective randomised clinical trials cannot. However, this work struggles with many of the same shortcomings of the broader machine learning studies in health-care literature. Foremost, what is the clinical utility? Predicted probability of mortality presents two options. The first is to alter the treatment plan when the probability of death continues to rise: perhaps the team needs to consider another source of infection or broaden antibiotic coverage. The second is to discuss with the patient or their family whether continued aggressive interventions in the face of increasing futility are within the patient’s goals of care. In both cases, the prediction of mortality at 90 days seems irrelevant at or near the time of ICU discharge. Knowing this probability is interesting from a technical, machine learning perspective, but perhaps less so in the aforementioned clinical decision making. Of note, the authors accrued data for their model with a setup similar to the SAPS III model for mortality prediction, but their comparison with SAPS III is limited because the latter predicts in-hospital mortality.

The authors also make a clear effort to show the generalisability of their system. They choose hyperparameters by cross validation, examine their model on a holdout test set, and then externally validate their final model on data from a separate centre in Denmark, in keeping with the TRIPOD guidelines.^4^ However, models that stand up to further scrutiny are the exception as opposed to the rule in clinical predictive modelling. With the known issues of algorithmic bias and the ongoing reproducibility crisis, the optimal way forwards is for all data and code to be released publicly for broader validation by the community. As the health-related data deluge continues and as the interest in data science grows, the reproducibility crisis is expected to worsen^5^ unless a more collaborative research ecosystem is created, as exemplified by initiatives such as the Medical Information Mart for Intensive Care^6^ and the Global Alliance for Genomics & Health.

But progress in machine learning, which has led to the emergence of new domains such as algorithmic bias, artificial intelligence explainability and ethics, and adversarial generative networks, has outpaced its adoption in health care. Despite thousands of publications and conference proceedings on medical algorithms, only a handful have been prospectively evaluated with clinical endpoints (rather than model performance) as outcomes.^7–10^ The few trials that have been published have not shown measurable improvement in population health. The machine learning community flocks to specialties where datasets exist—radiology, ophthalmology, critical care medicine—rather than in specialties where there is a dearth of randomised controlled trials, such as paediatrics, obstetrics and gynaecology, surgery, or primary care and mental health, where machine learning can have the biggest impact. With little input from providers at the frontlines of care, data scientists are sometimes working with a superficial understanding of health, attempting to predict disease trajectories and resource use that are clearly more heavily influenced by social determinants of health rather than the vital sign measurements, laboratory test results, and radiological images included in their models. Furthermore, the majority of models are being trained and validated on data from a handful of countries, further reinforcing a tradition that has historically skewed the generation of medical knowledge, uncovering medical truths that cannot be generalised to under-represented populations. A better roadmap is needed to leverage the value of machine learning and derive knowledge from the zettabytes of health data we collect in the process of care.
